# *Citrus* Flavones: An Update on Sources, Biological Functions, and Health Promoting Properties

**DOI:** 10.3390/plants9030288

**Published:** 2020-02-26

**Authors:** Davide Barreca, Giuseppina Mandalari, Antonella Calderaro, Antonella Smeriglio, Domenico Trombetta, Maria Rosa Felice, Giuseppe Gattuso

**Affiliations:** 1 Department of Chemical, Biological, Pharmaceutical and Environmental Sciences, University of Messina, Viale F. Stagno d’Alcontres 31, 98166 Messina, Italy; gmandalari@unime.it (G.M.); asmeriglio@unime.it (A.S.); dtrombetta@unime.it (D.T.); mrfelice@unime.it (M.R.F.); ggattuso@unime.it (G.G.); 2 Department of Agricultural Science, Università degli Studi Mediterranea, Feo di Vito, IT-89124 Reggio Calabria, Italy; anto.calderaro@gmail.com

**Keywords:** flavones, *Citrus*, antioxidant, antimicrobial, anticancer, anti-inflammatory

## Abstract

*Citrus* spp. are among the most widespread plants cultivated worldwide and every year millions of tons of fruit, juices, or processed compounds are produced and consumed, representing one of the main sources of nutrients in human diet. Among these, the flavonoids play a key role in providing a wide range of health beneficial effects. Apigenin, diosmetin, luteolin, acacetin, chrysoeriol, and their respective glycosides, that occur in concentrations up to 60 mg/L, are the most common flavones found in *Citrus* fruits and juices. The unique characteristics of their basic skeleton and the nature and position of the substituents have attracted and stimulated vigorous investigations as a consequence of an enormous biological potential, that manifests itself as (among other properties) antioxidant, anti-inflammatory, antiviral, antimicrobial, and anticancer activities. This review analyzes the biochemical, pharmacological, and biological properties of *Citrus* flavones, emphasizing their occurrence in *Citrus* spp. fruits and juices, on their bioavailability, and their ability to modulate signal cascades and key metabolic enzymes both in vitro and in vivo. Electronic databases including PubMed, Scopus, Web of Science, and SciFinder were used to investigate recent published articles on *Citrus* spp. in terms of components and bioactivity potentials.

## 1. Introduction

Over the past few decades there has been a significant shift in the general attitude toward the relation between food, diet, and wellbeing. Consumer awareness has greatly increased, and growing attention is being focused on the health benefits (in a broad sense, both in terms of positive effects and of disease prevention) that can be gained from what is commonly considered ‘healthy food’. As a result, functional foods and nutraceuticals have progressively hit the market, meeting the growing demand for more and more exotic foods, complex preparations, or glamorous concoctions. Research on the composition of these food matrices, as well as on the beneficial effects their consumption may gift the consumer, fueled the powerful growth of the nutraceutical market. Technical improvements in analytical techniques also led to a much deeper knowledge on the composition of food, allowing researchers (and, from there, consumers) to better understand the subtle difference in effects and efficiency displayed by fresh or processed food, dietary supplements, and individual compounds upon intake.

**Figure 1:**
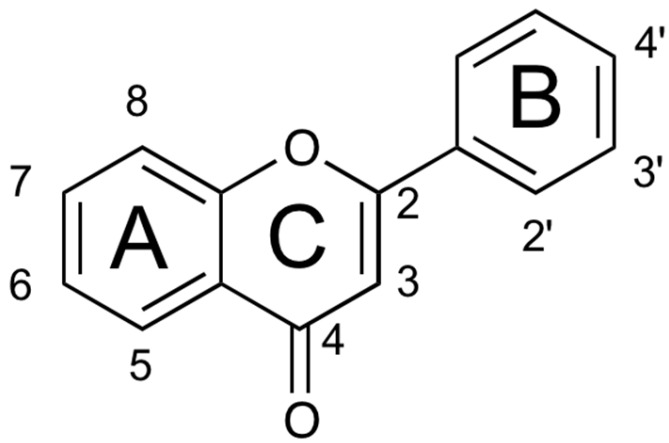
Structure of the 2-phenyl-1,4-benzopyrone.

However, albeit much effort has been devoted to the study of little-known products, several studies have kept their focus on those food products that have been for centuries the backbone of traditional medicine, well aware that the efficacy of traditional herbal remedies often derives from the bioactive compounds coming from the original plant/fruit material. Among these ‘classical’ sources, *Citrus* fruits, juices, and related products definitively stand out, owing to the fact that they are grown almost everywhere and they are commonly part of the diet people from the vast majority of countries and cultures. As a matter of fact, current worldwide production of oranges, mandarins/tangerines, grapefruits, and lemons/limes is estimated at about 94 million tons, with oranges alone totaling half of that figure [1]. However, citrus crops currently grown are not limited to those mainstream species. The genus *Citrus* (family: Rutaceae, subfamily: Aurantioideae) is fairly variegated, comprising a large number of species, varieties, cultivars, and hybrids. It has been classified according to two different systems: Swingle firstly defined 16 species, further subcategorized into varieties and hybrids. Tanaka later reorganized the entire genus into 156 species, allotting most varieties and cultivars their own species. However, *Citrus* taxonomy is not straightforward, given how prone they are to natural hybridization [2,3,4,5]. In fact, the species currently grown are the product of spontaneous or man-made cross-pollination from few ancestral species among which, according to recent phylogenetic, genomic, and biogeographical studies, the most ancient are considered to be *Citrus medica* (citron), *Citrus reticulata* (tangerine) and *Citrus maxima* (pummelo) [6]. The Authors of this study went as far as proposing a region that includes the eastern area of Assam, northern Myanmar, and western Yunnan as the place of origin of the first *Citrus* species.

*Citrus* fruits and juices are one of the main sources of nutrients in a standard balanced diet [7]. Leaving aside ascorbic acid, one of the main groups of compounds responsible for many of the health-beneficial effects are the (poly)phenolics, with special reference to the flavonoids [8].

The flavonoid family formally descends from the basic skeleton of 2-phenyl-1,4-benzopyrone (Figure 1), but the different flavonoid classes display many structural differences that, along with multiple substitution on the two aromatic and on the heterocyclic ring, account for the more than 8000 flavonoid derivatives isolated so far [9]. They are secondary metabolites, ubiquitous in the plant kingdom (especially green plants), and their biosynthesis occurs via the shikimate pathway and the phenylpropanoid metabolism [10]. They play several roles in plants, providing protection from different biotic and abiotic stress. They also act as a UV-filter, as signal molecules, and as antimicrobial defensive compounds [11]. Furthermore, they are responsible for the color and aroma of fruits and flowers, attracting pollinators, and protecting the plant against frost and drought.

**Figure 2:**
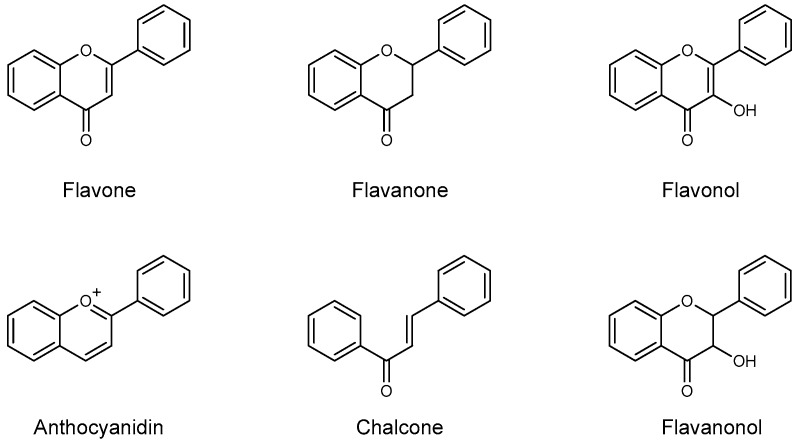
Core skeleton of the flavonoid subclasses generally found in *Citrus* fruits and juices.

Based on the differences on the phenylbenzopyrone core, flavonoids have been divided in many subgroups, which may have a very different distribution in plant species. In *Citrus* species, however, there are few recurring classes that are consistently found as the main components: the flavanones, the flavones, the flavonols, and to a minor extent the anthocyanidins, the flavanonols, and the chalcones (Figure 2). Flavones possess the 2-phenyl-1,4-benzopyrone skeleton, with hydroxyl groups usually (but not only, vide infra, Table 1) at the 5- and 7-position, and often also at the 4’- and/or 3’-position. Many different flavones have been isolated in *Citrus* fruits and juices, although they are often minor components of the flavonoid fraction. Even though they can be found in their aglycone form, they are often seen substituted with mono- or disaccharide moieties at some or all of the OH groups, as well as at the 6- or 8-position with a *C*-glycosidic bond. Among flavones, polymethoxyflavones (PMFs) may probably deserve their own subgroup, as they are a group of compounds bearing usually up to 5–7 OMe and OH groups in different positions [12] (Table 2). The flavones *O*- and *C*-glycoside found so far in *Citrus* juices are listed in Table 3.

Flavanones are generally the most abundant individual components of the flavonoid pool of *Citrus* fruits and juices [13]. They differ from flavones for the hydrogenation of the double bond in the 2-position of the A ring. As the flavones, they bear OH groups at the 5-, 7-, and 4’-position (sometimes in the 3’-position as well). They often occur as their glycosylated form, with the disaccharides rutinose (α-1,6-L-rhamnosyl-D-glucose) and neohesperidose (α-1,2-L-rhamnosyl-D-glucose) being quite common.

Flavononols and flavanols are related to the previous two groups for the presence of a hydroxyl group at their 3-position. Anthocyanidins are strongly pigmented compounds and appear only in brightly (red, purple) colored citrus fruits, such as blood oranges, to name the most common example [14,15]. Chalcones and their 2,3-dihydro derivatives are less common, although in some noticeable cases they are present in high amount (e.g., kumquat [16]).

## 2. Techniques of Identification and Quantification of Flavones in *Citrus* Varieties

The main techniques for identification and quantification of *Citrus* flavones are based on high performance liquid chromatography (HPLC) coupled with DAD detection, without preliminary derivatization of the starting sample, and MS or MS-MS system and ^1^H NMR spectroscopy [17,18,19,20,21,22,23]. In order to obtain a good yield in the extraction process, the choice of the appropriate solvent of extraction is of fundamental importance, in connection also to the starting natural matrix to be extracted [13,17,18,21,22,23]. In fact, the solubility of these compounds is generally not very high. Good extraction solvents are ethanol or methanol (absolute or mixed with low percentages of water), or dimethylsulfoxide.

The presence of flavone derivatives can be initially screened by the analysis of UV-visible spectra of the chromatographic peaks obtained with a DAD detector, characterized by the presence of two well defined bands at around 280 and 320 nm, and by the presence of shoulders in the proximity of these two bands determined by the presence of different substituents at rings A and B [22]. Almost all the flavones identified in *Citrus* are in their glycosylated forms and of particular interest is the application of MS and MS-MS identification techniques to obtain unequivocal information on the tested compounds. In fact, the MS and MS-MS spectra, both in positive and negative ion mode, of *Citrus* flavone glycosides have characteristic fragmentation patterns, strongly dependent on the number or the nature of the saccharide moieties and their *C*- or *O*-glycosidic linkages [22]. 

As mentioned above, after flavanones, flavones are the second most abundant class of flavonoids found in *Citrus*. Taking into account that citrus are commonly consumed as fresh fruits, or as hand-squeezed or industrially processed juices, most investigations (as well as the present survey) have been focused on fruit or juice content, especially for widespread species such as lemons, sour orange, bergamots, blond or blood oranges, mandarins, grapefruits, limes, kumquats, tangerines, chinotto, and tangors. 

Flavone contents of samples from these fruits, reported in Table 4, Table 5 and Table 6, range from trace amounts to 40–60 mg/L in the richest species [14,15,16,19,20,22,24,25,26,27,28,29,30,31,32,33,34,35]. Overall, in most citrus juices, vicenin-2 is, by far, the most abundant flavone derivative, with the highest level in bergamot cultivars (Fantastico, Femminello and Castaganaro), with values ranging from 44 to 55 mg/L in the juice, followed by blond and blood orange with 20–37 mg/L and tangelo with 2–4 mg/L. Additionally, other *C*-glycosyl flavones (lucenin-2, stellarin-2, isovitexin, scoparin, and lucenin 2,4-methyl ether) are present in a significant amount in the *Citrus* spp. (Table 4, Table 5 and Table 6), but in a much lower amount [19,22,25,26,27,28,29,30,31,32,33,34,35,36,37,38,39,40]. Rhoifolin, diosmin, and neodiosmin are the main flavone *O*-glycosides identified with values ranging from ≈2 to ≈30 mg/L (Table 4, Table 5 and Table 6). In addition, polymethoxy flavones are present in commonly consumed citrus juices, especially in the commercial products, but are almost completely absent in the hand-squeezed juice or in the peeled fruit pulp. In fact, these lipophilic compounds are mainly located in the peel, where they are components of the essential oil fraction. Flavones are present also in the leaves, in the seeds, and in the cortex, but obviously the main sources of flavones for human nutrition are the fruits and their juices.

## 3. Absorption Uptake and Pharmacokinetics

Dietary flavones are mainly glycosides and, from this point of view, their fate is determined by how and where they are absorbed, metabolized, transported, and excreted. The absorption of orally ingested *O*-glycosyl flavones and aglycones has been the subject of several in vivo studies, particularly in rats. Most of them have shown that apigenin, luteolin, and their simple glycosides are absorbed quickly. The time to reach the maximum plasma concentration (Tmax) [36] was generally ≤ 1 h, with a maximum plasma concentration (Cmax) between 1 and 100 μmol/L [37], depending on dose and type of food consumed (matrix effect). As for other food glycosylated flavonoids, flavones must be hydrolyzed before absorption [38], to be then metabolized into glucuronides or sulphates, before reaching the systemic circulation. To date, it is still not clear what the role of the stomach is into the absorption of flavones in humans. Although few compounds are absorbed into the stomach due to the relatively small surface area [37], several ex vivo models show how glycosides can be absorbed and hydrolyzed by stomach-specific β-glycosidase. The small intestine, which has a larger surface than the stomach and contains villi and microvilli, is much more effective in absorbing compounds such as flavones [37]. Furthermore, there is experimental evidence showing that the small intestine is an important site of hydrolysis and metabolism of *O*-glycosyl flavones. Flavonoids hydrolyzed by β-glycosidase in the small intestine were tested in vitro on Caco-2 models. Two β-glucosidases within small intestine cells can cleave flavonoid glycosides, with the greatest effect on flavonol, flavone, flavanone, and isoflavone *O*-glycosides [37,39]. The one that most likely acts on flavone glycosides in the small intestine is the lactase-phlorizin hydrolase, a membrane bound enzyme. The second one is a cytosolic β-glucosidase (CBG) present within intestinal cells, which also resides in the cytosol of liver, spleen, and kidney cells. Before hydrolyzation by CBG, glycosidic flavonoids must first be actively or passively transported to the cytosol. Some flavonoids such as quercetin are transported into the cells by passive diffusion [40]. Moreover, although the sodium-dependent glucose transporter 1 is involved in the uptake of some flavonoid glycosides such as quercetin 3-*O*-glucoside [41], it seems that this mechanism is not involved in the transport of flavones because some of them, such as luteolin, specifically inhibit this transporter [42]. In light of this, the transport of less polar molecules (e.g., aglycone) across the lipid membrane, seems to be favored [41]. In light of this, it is clear that flavones *O*-glycosides must be hydrolyzed to the respective aglycons before absorption in the small intestine epithelial cells can take place and, therefore, CBG being a cytosolic enzyme, it does not play a role in their deglycosylation. After that, aglycons or flavones *O*-glycosides metabolites are conveyed to the systematic circulation reaching the liver through the portal vein [37]. Phase II enzymes metabolize flavones in both the small intestine and liver. In particular, flavones are widely metabolized by the intestinal cells to glucuronidated and sulphated forms, which efflux back to the intestinal lumen and bloodstream. Multi-drug resistance-related proteins and organic anion transporters mediate the apigenin sulfate efflux, while only multi-drug resistance proteins mediate the outflow of apigenin glucuronide. 

The outflow of flavone metabolites is probably the limiting factor in transport through the intestinal membrane [37]. In vitro studies with human liver and intestinal microsomes have shown that luteolin is glucuronidated mainly in position 7 in hepatocytes and in positions 3’ and 4’ in enterocytes. This behavior is completely different in rat microsomes, where glucurono-conjugation occurs mainly in the 3’ position. When apigenin was incubated with microsomes and cytosol isolated from human liver and intestinal tissue, monoglucuronides and monosulfates were produced, respectively. Moreover, apigenin was more rapidly glucuronidated than sulfonated [43]. The metabolism of apigenin to luteolin has previously been reported in rats and it is mediated by the phase I enzyme cytochrome P450 [37]. This conversion has also been observed in human liver microsomes.

Polymethoxyflavones have attracted a lot of attention because of their high oral bioavailability with respect to hydroxyflavones. This is due to the lipophilic nature conferred to the molecule by the many methoxy groups [44]. However, it is well known that the bioavailability of these compounds depends on the location and the number of methoxy groups. Tangeretin and nobiletin, together with other PMFs present in the peel of *Citrus* fruits, show different chemical and physical features compared to other flavonoids and these properties probably play a pivotal role in influencing the metabolism and pharmacokinetics of these compounds in animals. For example, tangeretin, a pentamethoxyflavone, shows a greater oral bioavailability in comparison with 5,7,3’,4’-tetramethoxyflavone in rats. This is due, at least in part, to its greater number of methoxy groups. Studies carried out on Caco-2 cells have shown that the permeability of PMFs is approximately 5–8 times higher than non-methylated flavones [44]. Regarding distribution, tangeretin is widely distributed after the administration of a single dose of 50 mg/kg body weight with a high organotropism. In rats, after oral administration, tangeretin concentrates in kidneys, lungs, and the liver. Demethylation and conjugation have been identified as the two main metabolic pathways of PMF [44]. In fact, in rat liver microsomes, tangeretin is metabolized to 4’-hydroxy-5,6,7,8-tetramethoxyflavone and 3’,4’-dihydroxy-5,6,7,8-tetramethoxyflavone [44]. Even at intestinal levels, the metabolism of PMF leads to the formation of demethylated metabolites by intestinal bacteria [45,46]. After 4 h by oral administration, only 24% of tangeretin was detected in the organs and digestive tract, indicating that approximately 76% of flavone is absorbed or retained in the lumen of the digestive tract as metabolites, such as demethylated derivatives [44]. Recent in vivo investigations have reported that the main PMF urinary metabolites were demethylated flavones, glucuronides, and sulphoconjugates, while only demethylated flavones were found in the feces.

Diosmin (diosmetin 7-*O*-rutinoside) and diosmetin are two natural bioflavonoids widely present in several *Citrus* fruits. After oral administration, intestinal microflora enzymes rapidly hydrolyze diosmin into its aglycone, diosmetin, which is then absorbed through the intestinal wall. At systemic circulation level, diosmetin is enzymatically esterified to its relevant metabolite, 3-*O*-glucuronide, which is further esterified into 3,7-di-*O*-glucuronide [47,48]. Experimental evidence has shown that diosmin has the ability to inhibit P-glycoprotein [45] and several cytochrome P450 (CYP) enzyme isoforms (CYP1A1, CYP1A2, CYP2B1, CYP2C8, CYP2C9, CYP2D6, CYP2E1, CYP3A4, and CYP3A5). Specifically, the inhibition of drug-biotransformation CYP 450 enzymes [49] influences metabolic and pharmacokinetic properties of themselves and other simultaneously administered drugs [50]. Literature data suggest that diosmin is poorly absorbed after oral administration and this affects all pharmacokinetic parameters, including bioavailability [51]. It has been also observed that plasma concentrations have improved slightly when diosmin was administered in a micronized form, as the smaller particle size provided an increased surface area available for intestinal absorption [52].

*C*-glycosides, as well as *O*-glycosides, show a lower bioavailability in humans than in rats. Luteolin or apigenin *C*-glycosides metabolites were found in plasma after human ingestion of rooibos tea, with concentration <1 nmol/L [53]. Analysis conducted on gastrointestinal and stool extracts revealed the formation of three main metabolites by intestinal bacteria: floroglucinol, hydrocaffeic acid, and floretic acid. When the luteolin 6-*C*-glucoside (isoorientin) was incubated with the human intestinal bacteria, a small amount of luteolin aglycone was present. However, the most abundant metabolites were 3,4-dihydroxyphenylpropionic acid and eriodictyol, together with small amounts of 6-*C*-glucosyl eriodictyol and floroglucinol [37].

## 4. Antioxidant and Anti-Inflammatory Activity of *Citrus* Flavones

*Citrus* flavones, among the identified secondary metabolites in *Citrus* spp., are the most useful and promising compounds with antioxidant and anti-inflammatory activity [18,22,54,55,56,57,58,59]. They are characterized by a basic flavonoid skeleton, but present a well-defined set of substitutions and modifications which make them unique and with specific biological potential. The most common flavones identified in *Citrus* species are the glycosylated forms of luteolin, apigenin, diosmetin, acacetin, chrysoeriol, and isoscutellarein. In their glycosylated forms they generate *O*- and *C*- derivatives, whose properties are rather different. In general, the antioxidant efficiency of flavonoids depends on the aglycone structure and on the presence and position of hydroxyl groups, which in turn determine the extent of stabilization of the phenoxy radicals that originates from the interaction of the flavonoid with a radical [60,61,62,63,64,65,66,67,68]. Differently from other flavonoid classes (noticeably the flavanones), the flavones are characterized by the key presence of the C2-C3 double bond (conjugated with the carbonyl group at the 4-position), which puts in conjugation rings A and B, thus allowing a much greater electron delocalization of the radicals resulting from the extraction of H^•^ from an OH group [69,70,71,72,73]. Luteolin, for instance, possesses all the structural elements required to perform as an antioxidant: besides the presence of the carbonyl-conjugated C2-C3 double in ring C, it features an *ortho*-dihydroxy group (that is, in a catechol-like arrangement) on the B ring and a hydroxyl group at C5 of ring A. Apigenin has a good efficiency as well, even though it differs from luteolin for the absence of the OH group at the 3’ position of ring B, whereas isoscutellarein compensates the absence of the same 3’-OH group with the presence of catechol like-substitution pattern on the A-ring. Additionally, diosmetin and chrysoeriol show good activity, but they are generally less efficient than luteolin for the presence of a methoxy group at C4’ or C3’, respectively, which decreases the antioxidant potential [74]. Moreover, in general, *O*-glycosylated derivatives show much lower antioxidant activity than the corresponding aglycones, owing to the fact that a good part of the radical quenching mechanism of flavones relies on the presence of free OH groups [75]. 

Inflammation is an important aspect in several diseases (such as cardiovascular, cancer, asthma, diabetes) and citrus flavonoids, introduced with diet, have different effects on inflammation acting at different stages with remarkable influence on several inflammatory diseases. Along with the antioxidant activity described above, *Citrus* flavonoids—and in particular flavones—inhibit regulatory enzymes or transcription factors important for controlling mediators involved in inflammation, with the potential to inhibit the onset and development of inflammatory diseases, in both in vitro and in animal models. In the study of Kimata et al. [76] luteolin showed to significantly decrease (in a concentration-dependent manner) the release of histamine, leukotrienes, prostaglandin D2, and granulocyte macrophage-colony stimulating factor from human mast cells in culture. Moreover, luteolin strongly inhibits and suppresses Ca^2+^ influx and PKC translocation and activity, extracellular signal-regulated kinases and c-Jun NH_2_-terminal kinase, while it has no effect on activation of p38 mitogen-activated protein kinase pathway. In 2015, Weng et al. [77] described the influence of luteolin and of its modified structural analog (methlut) in the release of mediators implicated in asthma. The results obtained on human mast cells showed an inhibition of histamine, β-hexosaminidase, TNF, and CCL2 release in mast cells derived from human cord blood, elements implicated in the recruitment of different inflammatory cells, with the block, at gene and protein levels, of intracellular calcium release and/or inhibition of NF-κB [77]. Moreover, in rat heart after ischemia/reperfusion, luteolin ameliorates systolic/diastolic functions increasing the activity of sarcoplasmic reticulum Ca^2+^-ATPase via the activation of the p38 MAPK pathway [78].

Funakoshi-Tago et al. [79] have analyzed the mechanisms implicated in the anti-inflammatory activity of apigenin, luteolin, and fisetin, based on their related structure, and found both significant similarity and difference in the modulation of biological pathways and proteins. They inhibit TNFα-induced NF-κB transcriptional activation, although IκB proteins and the nuclear translocation and DNA binding activity of NF-κB p65 are not influenced by their action. The flavones direct their action on the inhibition of transcriptional activity of GAL4-NF-κB p65 fusion protein and of the transcriptional activation of NF-κB. Moreover, apigenin and luteolin had low effects in the inhibition of TNFα-induced JNK activation, while they had no effect on TNFα-induced activation of ERK and p38. The activity of apigenin and luteolin (but not fisetin) is evident also in the inhibition of TNFα-induced expression of CCL2/MCP-1 and CXCL1/KC. Based on the above reported activities the administration of apigenin and luteolin resulted in a remarkable inhibition of acute carrageenan-induced paw edema in mice.

## 5. Antimicrobial and Antiviral Effect of *Citrus* Flavones

The antimicrobial and antiviral potential of polyphenols has been widely investigated. Polyphenols extracted from almond skins showed a remarkable antimicrobial potential against Gram-negative bacteria (*Escherichia coli, Pseudomonas aeruginosa, Salmonella enterica, Serratia marcescens*), Gram-positive bacteria (*Listeria monocytogenes, Enterococcus hirae, Staphylococcus aureus, Enterococcus durans*) and the yeast *Candida albicans* [80]. In another study, the effect of polyphenols contained in almond skins was evaluated against cagA-positive and -negative clinical isolates of *Helicobacter pylori* [81]. The same bioactive compounds present in almond skins were also able to inhibit HSV-2 and HSV-1 replication [82,83].

Regarding polyphenols from *Citrus* spp., the antimicrobial potential of extracts rich in flavonoids obtained from bergamot peel (*Citrus bergamia* Risso) was evaluated against Gram-negative bacteria (*Escherichia coli, Pseudomonas putida, Salmonella enterica*), Gram-positive bacteria (*Listeria innocua, Bacillus subtilis, Staphylococcus aureus, Lactococcus lactis*), and the yeast *Saccharomyces cerevisiae*. The extracts were mostly active against the Gram-negative bacteria, with minimum inhibitory concentrations in the range 200–800 mg mL^–1^ [84]. Bergamot juice was also tested against cagA-positive and -negative clinical isolates of *Helicobacter pylori*: the results confirmed that bergamot juice (2.5%, *v*/*v*) inhibited the growth of 50% of clinical isolates, whereas 5% (*v*/*v*) inhibited 90% and a synergistic effect was observed with the two antibiotics amoxicillin and metronizadole, both used for the treatment of *Helicobacter pylori* [85].

Given the increased problem of antimicrobial and antiviral resistance worldwide, food and pharmaceutical industries are looking into novel bioactives to be used alone or in combination with traditional drugs. Uckoo et al. [86] explored the activity of nine polymethoxyflavones and a limonoid extracted and identified from the less explored Miaray mandarin to inhibit cell–cell signaling and biofilm formation in *Vibrio harveyi*. The polymethoxyflavones 3,5,6,7,8,3’,4’-heptamethoxyflavone and 3,5,7,8,3’,4’-hexamethoxyflavone were the most powerful compounds able to inhibit biofilm activity. Bacterial biofilm is proven to be increasingly difficult to eradicate by antibiotic treatment, especially within hospital-acquired infections. The antibacterial potential of *Citrus* polymethoxyflavones has also been evaluated by Johann et al. [87] against standard strains of the Gram-negative bacteria *Escherichia coli* and the Gram-positive bacteria *Staphylococcus aureus*. The most potent compounds were 4’,5,6,7,8-pentamethoxyflavone (tangeretin) and 3’,4’,5,6,7,8-hexamethoxyflavone (nobiletin), both extracts from *C. reticulata*, and 6,7-dimethoxycoumarin (also known as escoparone, scoparone, or scoparin), extracted from *Citrus limon*. Nobiletin, extracted from *Citrus nobilis*, also showed an anti-inflammatory and antioxidant potential against generation of free radicals in an animal study, demonstrating a possible correlation between antioxidant and antimicrobial activity [88]. Additionally, an in-depth analysis was performed on the antimicrobial activity of a broad fraction obtained by preparative HPLC from tangelo (*Citrus reticulata* × *Citrus paradisi*) juice containing all the detected flavonoids, and of five subfractions characterized by the presence mainly of *C*-glycosyl flavonoids, *O*-triglycosyl flavanone, *O*-glycosyl flavones, *O*-glycosyl flavanones, and polymethoxyflavones, in terms of MICs for *Staphylococcus aureus* [29]. The broad fraction containing all the identified flavonoids showed an evident MICs only at 1.0 mg/mL, even though an influence on growth was already noticeable at 0.5 mg/mL and, among the five fractions, the one containing the polymethoxyflavones (sinensetin, nobiletin, and tangeretin) is responsible for 50% of this activity, while *C*-glycosyl and *O*-glycosyl flavones are responsible for less than 12%.

An interesting study has reported the synergistic effect of the combination diosmetin and the antibiotic erythromycin against methicillin-resistant *Staphylococcus aureus* (MRSA) [89]. The increasing prevalence of MRSA strains poses a serious public health issue, especially within hospital environments. MRSA strains, which are currently gaining resistance to novel antibiotics, are also prone to produce biofilms. Diosmin and diosmetin, detected in a variety of *Citrus* fruits, were tested against MRSA strains, either alone or in combination with erythromycin. The association experiments were performed by the checkerboard assay. The results showed that diosmetin, in association with erythromycin, was active against the ABC transporter overexpressed MRSA RN4220/pUL5054 and inhibited the MRSA pyruvate kinase. The antibacterial potential of peel and pulp extracts of *Citrus* samples from Aceh, Indonesia, was recently evaluated in vitro against *Klebsiella pneumoniae* and *Staphylococcus aureus* [90]. Amongst the polymethoxylated flavones identified in the Aceh samples, sinensetin and tangeretin were the most abundant. The results demonstrated bacteriostatic and bactericidal activity against both tested strains. Interestingly, the extracts were slightly more potent against *Staphylococcus aureus* than *Klebsiella pneumoniae* and the peel extracts exhibited slightly better activity than the pulp. 

Amongst other flavones widely distributed in fruits, plants, and spices, chrysin and apigenin showed moderate or low antimicrobial properties against *Escherichia coli*, *Pseudomonas aeruginosa*, *Enterococcus faecalis*, and *Staphylococcus aureus*, the Gram-negative bacteria being more susceptible to the natural compounds. Interestingly, the presence of hydroxyl groups in the phenyl rings A and B did not influence their activity, with the exception of *Staphylococcus aureus* [91]. On the contrary, Echeverría et al. [92] reported that methylation of one hydroxyl group decreased the activity in 3-*O*-methylgalangin, and methylation of both hydroxyl groups caused inactivation against *Bacillus subtilis* and *Escherichia coli*. These findings highlight the importance of the structure–activity relationship when considering the antibacterial potential of natural compounds. 

A number of studies have investigated the potential antiviral properties of *Citrus* flavones. Xu et al. [93] described the in vitro antiviral potential of a supercritical fluid extract of “Guangchenpi”, the edible and medicinal pericarps of *C. reticulata* ‘Chachi’, against the respiratory syncytial virus (RSV). RSV is a common pathogen responsible for pneumonia and bronchiolitis, which mainly affect infants and young children. Interestingly, the two most abundant polymethoxylated flavones identified in the extract of “Guangchenpi”, tangeretin and nobiletin, determined a dose-dependent inhibition of the virus, calculated by plaque reduction assay. The same authors have subsequently investigated the activity of tangeretin from *C. reticulata* in vivo using 3-week-old male BALB/c mice [94]. As previously shown in vitro, the results from this study confirmed an inhibition of the RSV replication exerted by tangeretin in the lung of the mice. This inhibition in the RSV replication also correlated with an important anti-inflammatory effect induced by tangeretin, by suppressing nuclear factor-κB (NF-κB) activation in the animal lungs. Tangeretin was also tested for its antiviral potential against Lassa virus, an Arenavirus causing hemorrhagic fever in humans: the results showed that the polymethoxylated flavone was able to block the entrance of the virus within the cells [95]. Amongst other flavones recently tested for their antiviral potential, ladanein (5,6,7-trihydroxylated flavone) exhibited a potent virucidal effect against a range of enveloped virus particles [96]. While the activity of ladanein had previously been confirmed against hepatitis C virus particles, the investigation reported on the impact of iron complexation and medium acidity on the antiviral effect. Moreover, a recent docking study show the potentiality of flavones to inhibit multiplication of viruses of different influenza serotypes [97]. The results demonstrated that substitution by fluorinated groups determined a significant improvement of the antiviral effect, whereas the combination with a strong iron chelator produced the loss of the antiviral activity. These findings allow a deeper insight into the structure–function relationships of bioactive analogues, as well as composition of the growth medium, all essential in the antiviral activity of the flavones.

Taken all together, these considerations share some light on the possible applications of *Citrus* flavones as potential antibacterial and antiviral candidates, in order to fight world-wide resistance. Further development and improvement of novel antibacterial and antiviral drug candidates need to be established in order to formulate drugs for topical applications. It is also important to evaluate the structure–function relationships amongst the natural bioactives as well as their potential synergistic interactions with widely used drugs.

## 6. Anticancer Activity of *Citrus* Flavones

Flavones present in *Citrus* spp. show promising anticancer activity, especially as far as apigenin, luteolin, diosmetin, and chrysoeriol are concerned, due to their remarkable antioxidant activity, capacity to decrease proliferative growth, activation/blocking of metabolic pathways, and key enzymes, arrest of the cell cycle, reversal of multidrug resistance, inhibition of angiogenesis, activation of apoptotic events, or a combination of these mechanisms [98,99]. Moreover, the inhibition of protein kinase and antiproliferation activity seem specifically linked to the hydroxylation pattern of the B ring of the flavones (such as luteolin) and several flavones remarkably linked to cell surface signal transduction enzymes (such as, for instance, focal adhesion kinases, and protein tyrosine kinases). The mechanisms underlying the potential anticancer action of flavones are complex and enclose structural and molecular aspects and further studies are necessary in order to develop a plant flavone-based strategy.

### 6.1. Apigenin

Apigenin has been the object of intense studies in the last 40 years and among the different beneficial effects for organisms, its activity with respect to different kind of cancers is especially interesting. This feature was nicely described in a recent review [56]. This activity is mainly linked to its antioxidant effect, its capacity to decrease proliferative growth, the arrest of the cell cycle, and its proapoptotic properties [100,101,102,103,104,105,106]. In the last period, further studies have tried to increase the set of information related to mechanism of action, using esophagus, breast, bronchial epithelial, NSLC (non-small cell lung cancer), and colon cancer cell lines. An in silico study has highlighted 125 potential target proteins of apigenin and most of them are related to inflammation response, antioxidant activity, and development of different types of cancer, confirming and, in part, extending the overall knowledge about the pathways in which apigenin can be involved [107].

In some tumors a great concentration of tumor necrosis factor α (TNF α) protein was found and transcriptomic analysis has demonstrated that apigenin is able to downregulate half of the genes that normally are upregulated by TNFα, all related to tumor promotion, invasion, and metastatic growth [108]. Interleukin-6 (IL-6) is another protein whose expression is negatively regulated by apigenin and the reduced expression affects not only tumor proliferation, but also migration and invasiveness [109]. Furthermore, to regulate TNFα and IL-6 production, apigenin seems to exert its anticancer activity also by decreasing expression of histone deacetylase, SIRT6, in a dose-dependent manner. This results in a minor viability of cells and a decrease of colony forming capacity [110]. Moreover, apigenin is able to reduce the inflammation effects of cigarette smoke, reducing ROS production by a mechanism that involves a decreased expression of miR-21, an onco-miRNA overexpressed in different types of cancer, and interleukin-8, a proinflammatory cytokine [111]. Another target of apigenin is BCRP (breast cancer resistance protein), one of the principal efflux transporter proteins, expressed in different organs and tissues and, in particular, it is overexpressed in different types of cancer where it seems to be involved in multidrug resistance (MDR) phenotypes. The co-administration of chemotherapeutic molecules and apigenin significantly reduces the efflux of drugs increasing their effectiveness [112]. Apigenin was also effective in cisplatin-resistant cancer cells as it is able to increase the apoptosis and autophagy rate probably by a mechanism in which mTOR pathway activity is reduced [113]. Another typology of cancer, in which apigenin is active, is a resistant form to different chemotherapeutic molecules, such as ABT-263 and AZD9291, because of epithelial growth factor receptor mutations (EGFRm). Mechanistic studies have demonstrated that co-administration of apigenin and ABT-263 causes a decrease of AKT and ERK phosphorylation rate that reflects on FoxO3a transcription factor activity and, consequently, on the transcription of a member of Bcl-2 family protein, Noxa, that in turns activate caspase-3 [109]. Apigenin was also used in vivo in a xenograft mouse model and a reduction of tumor volume and weight was noted, confirming its potential role as a chemotherapeutic molecule [109,113,114,115].

### 6.2. Luteolin 

Luteolin was extensively used as anticancer molecule in different studies and its efficiency was demonstrated both in vitro and in vivo. Considering different typologies of cancer, it is able to reduce the tumor size, growth rate, and vascularization, as described by Ahmed et al. [116], Imran et al. [117], and Avila-Carrasco et al. [118] in recent reviews, mainly by a mechanism in which apoptosis of cancer cells is induced. 

Given the potential of luteolin as chemotherapeutic agent, different studies are currently focusing on different types of tumors such as breast cancer, NSLC, squamous carcinoma, melanoma, colon cancer, and glioblastoma, using principally cancer cell lines for a better understanding of its mechanism of action. In every in vitro study, an increase of ROS production was seen, along with a decrease of cell survival, induction of apoptosis, and decrease of mobility and invasive abilities, even if different mechanisms are involved. Among these, a reduction of matrix metalloproteinase (MMP)-9 expression, a protein that is involved in extracellular matrix (ECM) degradation, was observed [119]. Moreover, MMP-2 can also be downregulated due to a decrease of phosphorylated AKT, and PI3K proteins [120]. Another target of luteolin is the catalytic subunit of telomerase, hTERT, that is downregulated such as its transcriptional inducer, c-myc, by a mechanism in which the cytoplasmic inactive form of NF-kB is stabilized. As result, the progressive shortening of telomeres induces apoptosis [121]. Luteolin can also regulate IL-6 production, by binding competition to IL-6Rα receptor, that causes the reduction of signal transducer and activator of transcription 3 (STAT3) phosphorylation, responsible for the transcription of different genes involved in cancer initiation and progression [122]. Another protein that is negatively regulated by luteolin is AIM2 (absent in melanoma 2), a central protein involved in the formation of inflammasome complex and inflammation, that leads to a decreased expression of caspase 1 and interleukin-1β, and increased expression of the p21 inhibitor cyclin kinase [123]. The expression of S100A7 protein (S100 calcium-binding protein) is also altered by luteolin; in fact, its expression is decreased, negatively influencing migration ability and invasiveness [124]. 

The involvement of post-transcriptional regulation by miRNA was also observed. In particular, luteolin increases expression of miR-203 and this alters the RAS/RAF/MEK/ERK pathway [125]. Moreover, luteolin-mediated upregulation of miR-384 reduces the expression of pleiotrophin, a protein that was found to be highly expressed in different types of tumors, influencing invasion and angiogenesis [126]. The expression of Nrf2 (nuclear factor erythroid 2-related factor 2) transcription factor, a protein very important in the detoxification of ROS, is also negatively regulated by luteolin and this causes an increase of intracellularly ROS concentration and apoptosis rate [127]. In contrast with these results, in another study an epigenetic mechanism of regulation was discovered; in fact, luteolin causes a reduction of ROS production, linked essentially to an increase of expression of p53 protein, and total and phosphorylated Nrf2. The high expression of Nrf2 seems to be linked to promoter DNA methylation that is high in cancer cells while it is reduced after administration of luteolin. In support of this, it was observed an increased expression of DNA demethylases, TET (ten eleven translocation) enzymes, combined with TET1 physical interaction with Nrf2 promoter. The interaction between Nrf2 and p53 proteins seems to be important for the induction of apoptosis [128].

The efficiency of luteolin as an anticancer molecule was also investigated in vivo by a xenograft mouse model of NSLC [123], Solid Ehrlich carcinoma, a model extensively used to study chemotherapeutic properties of molecules [129] and melanoma [126]. In all cases, it was found that luteolin is able to decrease tumor volume and weight by the same mechanisms observed in in vitro studies. A strategy for increase the bioavalibility of luteolin was to encapsulate the flavone in folic acid-modified poly(ethyleneglycol)-poly(caprolactone) (Fa-PEG-PCL) nano-micelles that make luteolin much more efficient with respect to when it is in its free form, reducing the concentration needed to kill cells, such as in glioblastoma. The same effect was also reported when xenograft mice were used [130].

### 6.3. Diosmetin

Diosmetin is another flavone whose use as an anticancer molecule is more recent with respect to the others. In the last years, its activity was assayed on different types of cancer cells such as breast cancer, hepatocellular carcinoma, melanoma, colon cancer, acute myeloid leukemia, prostate cancer, NSLC, and radioresistant lung cancer. These studies have highlighted that diosmetin is converted into luteolin and another product not characterized by cytochrome P450 CYP1 enzymes [131,132,133]. In all in vitro studies, diosmetin administration led to an increase of ROS production, to the arrest of the cells in G0/G1, G1/S, or G2/M phase, dependent on the typology of cancer cell model used, and induction of apoptosis pathway in a dose-dependent manner [134,135,136,137]. The mechanisms influenced by diosmetin are different and, probably, cell specific. An effect exerted by diosmetin is the upregulation of p53 protein and decreased expression of transforming growth factor-β (TGF- β), that is normally upregulated in cancer cells and is involved in regulation of extracellular matrix proteins’ expression [138]. In addition to the increase of p53 protein expression, diosmetin reduces NF-kB activity through the decrease of the expression of Notch3 receptor, a protein highly expressed in tumors [139]. Moreover, the mammalian target of rapamycin (mTOR) pathway seems to be also negatively influenced by diosmetin and this leads to an increase of autophagosomes formation, strictly correlated with an increase of apoptosis and decrease of cell survival rate [140]. Another pathway negatively influenced by diosmetin is BMP (bone morphogenetic proteins) dependent signaling that causes p21 protein upregulation [135]. Moreover, it inhibits DNA repair mechanisms by negative modulation of the AKT signaling pathway [132]. Another mechanism, used by diosmetin to control cell fate, is the binding to estrogen receptor-β (ERβ), a protein associated with antiproliferative activity, which leads to an increase in TNFα production [141]. The expression of CDKs and cyclins proteins is also negatively influenced by diosmetin by a mechanism in which reduced expression of c-myc protein leads to major expression of FOXO3a and p27 protein, an inhibitor of CDKs [136]. As for luteolin, diosmetin treatment reduces the steady state level of Nrf2 protein, by a mechanism in which the PI3K/AKT/GSK-3 pathway is involved with a consequent increase of ROS concentration [142]. Diosmetin is also able, in vitro, to decrease cellular migration and invasion abilities, inhibiting metastasis formation, by a mechanism in which the flavone decreases the expression of MMP-2 and MMP-9 proteins by reducing the MAPK pathway activity [143]. In in vivo studies, using a xenograft mouse model, diosmetin administration leads to decrease of tumor volume and weight, by the same mechanisms discovered in vitro [141,142,143,144]. Moreover, a reduction of tumor vascularization by a mechanism involving negative modulation of angiopoietin-2, a protein that contributes to the destabilization of endothelial cell–pericyte interactions, was also observed. As result, diosmetin influences the structure and functionality of vessels, restoring tumor blood flow through the tumor [145].

### 6.4. Chrysoeriol

The anticancer activity of chrysoeriol is less characterized and few studies are present in the literature. Its activity can be directed against key proteins involved in the initiation and progression of cancer such as CYP1B1, an enzyme that hydrolyzes β-17-estradiol (E2) to 4-hydroxy-E2 that is highly carcinogenic [146], or CK2 that is a constitutive protein kinase involved in different cellular functions whose concentration was found to be very high in different tumors, and inhibits apoptosis [147]. Another important protein, whose function can be regulated by chrysoeriol, is BCRP; in fact, the flavone is able to decrease drastically its efflux activity, reducing multidrug resistance typical of cancer cells [148]. Docking experiments and enzymatic assays have demonstrated that chrysoeriol is able also to bind and inhibit two kinases, VEGFR2 (vascular endothelial growth factor 2) and c-Met, both important for tumor progression and resistance to drugs [149]. 8-chrysoeriol, a derivate of chrysoeriol, can promote apoptosis by binding to the BH3 domain of Bcl2 antiapoptotic proteins, that are highly expressed in cancer cells, blocking their activity and inducing cells death [150]. Only few mechanistic studies were carried out, using breast cancer, myeloma, and lung carcinoma cell lines. In all cases, an increase of apoptosis rate and decreased proliferation was found. A downregulation of MMP-9 and COX2 (cyclooxygenase 2) expression dependent on the NF-kB pathway [151] and reduced activity of the PI3K/AKT/mTOR [152] and MAPK/ERK pathways [153] was observed. Moreover, an in vivo study has demonstrated that chrysoeriol can inhibit tumor growth and volume [153].

## 7. Conclusion and Future Perspectives

*Citrus* spp. flavones show promising, interesting, and useful biological properties with potentially a wide range of physiological and pharmacological effects, more important because they have been a part of human diet since the beginning of mankind. The data obtained in in vitro and in vivo laboratory investigations clearly demonstrates the antioxidant, anti-inflammatory, antimicrobial, and anticancer properties of these secondary metabolites, but epidemiologic and well-designed prospective human studies will be necessary in order to clarify the extent of their effects. However, light has to be shed on the mechanism of action of these fascinating polyphenolics from fruit to the human body, from the specific influences of the microstructural components in the release of these phytonutrients from their food matrix, down to the ability of these bioactive compounds, once released and absorbed, to activate and modulate complex processes inside the organism. Furthermore, efforts should be devoted to the study of new metabolites generated by internal microbiota, that may result in the production of new molecules that are sometimes more active than the parent compounds. These studies will pave the way to the development of new functional foods and nutraceuticals with health-promoting properties.

## Figures and Tables

**Table 1:** Flavone aglycones occurring in *Citrus* spp.

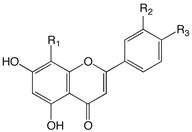			
Compound Name	R_1_	R_2_	R_3_
Acacetin	H	H	OMe
Isoscutellarein	OH	H	OH
Luteolin	H	OH	OH
Apigenin	H	H	OH
Diosmetin	H	OH	OMe
Chrysoeriol	H	OMe	OH

**Table 2:** Polymethoxyflavones occurring in *Citrus* spp.

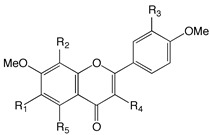					
Compound Name	R_1_	R_2_	R_3_	R_4_	R_5_
Quercetogetin	OMe	H	OMe	OMe	OMe
3,3’,4’,5,6,7,8-Heptamethoxyflavone	OMe	OMe	OMe	OMe	OMe
Natsudaidain	OMe	OMe	OMe	OH	OMe
Nobiletin	OMe	OMe	OMe	H	OMe
Sinensetin	OMe	H	OMe	H	OMe
Tangeretin	OMe	OMe	H	H	OMe
Tetramethylscutellarein	OMe	H	H	H	OMe
5-Hydroxy-3,7,3’,4’-tetramethoxyflavone	H	H	OMe	OMe	OH
3,3’,4’,5,7,8-Hexamethoxyflavone	H	OMe	OMe	OMe	OMe
Pentamethylquercetin	H	H	OMe	OMe	OMe
3’,4’,5,7,8-Pentamethoxyflavone	H	OMe	OMe	H	OMe
4’,5,7,8-Tetramethoxyflavone	H	OMe	H	H	OMe
4’,5,7-Trimethoxyflavone	H	H	H	H	OMe

**Table 3:** Glycosylated flavones occurring in *Citrus* spp.

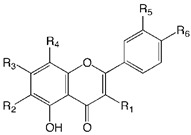						
Compound Name	R_1_	R_2_	R_3_	R_4_	R_5_	R_6_
Luteolin 6,8-di-*C*-glucoside (Lucenin-2)	H	Glu	OH	Glu	OH	OH
Apigenin 6,8-di-*C*-glucoside (Vicenin-2)	H	Glu	OH	Glu	H	OH
Chrysoeriol 6,8-di-*C*-glucoside (Stellarin-2)	H	Glu	OH	Glu	OMe	OH
Diosmetin 6,8-di-*C*-glucoside (Lucenin-2 4’-methyl ether)	H	Glu	OH	Glu	OH	OMe
Apigenin 7-*O*-neohesperidoside-4’-glucoside (Rhoifolin 4’-glucoside)	H	H	*O*-Nh	H	OH	*O*-Glu
Chrysoeriol 7-*O*-neohesperidoside-4’-glucoside	H	H	*O*-Nh	H	OMe	OH
Apigenin 6-*C*-glucoside (Isovitexin)	H	Glu	OH	H	H	OH
Luteolin 7-*O*-rutinoside	H	H	*O*-Ru	H	OH	OH
Chrysoeriol 8-*C*-glucoside (Scoparin)	H	H	OH	Glu	OMe	OH
Diosmetin 8-*C*-glucoside (Orientin 4’-methyl ether)	H	H	OH	Glu	OH	OMe
Apigenin 7-*O*-neohesperidoside (Rhoifolin)	H	H	*O*-Nh	H	OH	OH
Apigenin 7-*O*-rutinoside (Isorhoifolin)	H	H	*O*-Ru	H	OH	OH
Chrysoeriol 7-*O*-neohesperidoside	H	H	*O*-Nh	H	OMe	OH
Diosmetin 7-*O*-rutinoside (Diosmin)	H	H	*O*-Ru	H	OH	OMe
Diosmetin 7-*O*-neohesperidoside (Neodiosmin)	H	H	*O*-Nh	H	OH	OMe
Acacetin 3,6-di-*C*-glucoside	Glu	Glu	OH	H	H	OMe
Apigenin 8-*C*-neohesperidoside	H	H	OH	Nh	H	OH
Acacetin 8-*C*-neohesperidoside (2’’-*O*-rhamnosyl cytisoside)	H	H	OH	Nh	H	OMe
Acacetin 6-*C*-neohesperidoside (2’’-*O*-rhamnosyl isocytisoside)	H	Nh	OH	H	H	OMe
Acacetin 7-*O*-neohesperidoside (Fortunellin)	H	H	*O*-Nh	H	H	OMe

Ru: Rutinose; Nh: Neohesperidose.

**Table 4:** Flavone content in chinotto (*Citrus myrtifolia* Raf.), sour orange (*Citrus aurantium* L.), sweet lemon (*Citrus limetta* Risso), kumquat (*Fortunella japonica* Swingle), and tangelo (*Citrus reticulata* × *Citrus paradisi*).

*Species*	Chinotto	Sour Orange	Sweet Lemon	Kumquat		Tangelo
				Ripe	Unripe	
*Flavone*						
Vicenin-2	0.58 ± 0.06	1.54 ± 0.15	0.37 ± 0.02	Traces	0.13 ± 0.01	3.89 ± 0.11
Diosmin	0.20 ± 0.02	0.15	0.39 ± 0.07	------	------	
Lucenin-2	------	0.12 ± 0.012	------	------	------	0.10 ± 0.012
Scoparin	------	------	0.10 ± 0.03	------	------	------
Lucenin-2 4’- methyl ether	------	0.45 ± 0.06	0.75 ± 0.05	Traces	Traces	------
Rhoifolin	0.11 ± 0.02	0.79 ± 0.08	0.15 ± 0.02	Traces	------	------
Neodiosmin	0.11 ± 0.01	------	------	------	------	------
Rhoifolin 4’-*O*-glucoside	------	0.36 ± 0.04	------	------	------	------
Orientin 4’-methyl ether	------	------	0.10 ± 0.04	------	------	------
Acacetin 3,6-di-*C*-glucoside	------	------	------	Traces	Traces	------
Apigenin 8-*C*-neohesperidoside	------	------	------	0.26 ± 0.03	0.81 ± 0.08	------
Acacetin 8-*C*-neohesperidoside	------	------	------	0.60 ± 0.06	1.32 ± 0.13	------
Acacetin 6-*C*-neohesperidoside	------	------	------	0.70 ± 0.07	1.77 ± 0.2	------
Acacetin 7-*O*-neohesperidoside	------	------	------	0.82 ± 0.08	2.72 ± 0.3	------
Sinensetin	------	------	------	------	------	0.06 ± 0.02
Tangeretin	------	0.08	------	------	------	0.08 ± 0.012
Nobiletin	------	0.2	------	------	------	------
	[25,28]	[26]	[27]	[16]	[16]	[22]

**Table 5:** Flavone content in bergamot (*Citrus bergamia* Risso) and blood orange (*Citrus sinensis* (L.) Osbeck) varieties.

*Species*	Bergamot			Blood Orange		
*Cultivar*	Castagnaro	Fantastico	Femminello	Moro	Sanguinello	Tarocco
*Flavone*						
Vicenin-2	47.5 ± 4.7	44.1 ± 4.4	55.2 ± 5.5	37.53 ± 0.40	36.2 ± 2.40	32.22 ± 0.34
Lucenin-2	2.2 ± 0.22	2.5 ± 0.25	3.3 ± 0.3	10.48 ± 0.56	4.5 ± 0.42	7.23 ± 0.34
Stellarin-2	0.6 ± 0.06	0.7 ± 0.07	1.1 ± 0.1	22.13 ± 2.27	6.46 ± 1.64	0.78 ± 0.02
Isovitexin	2.5 ± 0.25	2.2 ± 0.2	3.1 ± 0.3	------	------	------
Scoparin	5.4 ± 0.54	5.9 ± 0.6	9.1 ± 0.9	7.14 ± 0.88	7.43 ± 0.90	------
Lucenin-2 4’-methyl ether	25.4 ± 2.5	32.7 ± 3.3	62.8 ± 6.3	11.73 ± 1.10	0.70 ± 0.03	0.26 ± 0.01
Rhoifolin	28.9 ± 2.9	26.2 ± 2.6	22.8 ± 2.3	------	------	------
Neodiosmin	15.3 ± 1.5	80.0 ± 8.0	27.1 ± 2.7	------	------	------
Rhoifolin 4’-*O*-glucoside	1.2 ± 0.12	1.2 ± 0.12	1.3 ± 0.1	------	------	------
Orientin 4’-methyl ether	1.7 ± 0.2	1.9 ± 0.2	4.2 ± 0.4	------	------	------
Chrysoeriol 7-*O*- neoesperidoside	10.6 ± 1.1	9.3 ± 0.9	17.2 ± 1.7	0.79 ± 0.09	0.50 ± 0.07	0.23 ± 0.01
Chrysoeriol 7-*O*- neohesperidoside -4′-*O*- glucoside	3.8 ± 0.4	4.1 ± 0.4	5.3 ± 0.5	------	------	------
	[22,24]	[22,24]	[22,24]	[14]	[15]	[15]

**Table 6:** Flavone content in lumia (*Citrus lumia* Risso and Poit.)*,* sweet orange (*Citrus sinensis*), grapefruit (*Citrus paradise*), clementine (*Citrus clementina*), and mandarin (*Citrus reticulata*).

*Species*	Lumia	Sweet Orange	Grapefruit	Clementine	Mandarine
*Flavone*					
Vicenin-2	------	5.72 ± 2.02	1.30 ± 0.16	0.70 ± 0.09	11.4 ± 0.60
Diosmin	22.8 ± 0.84	0.09 ± 0.009	------	1.25 ± 0.51	------
Lucenin-2 4’-methyl ether	------	0.35 ± 0.14	------	0.30 ± 0.02	4.80 ± 0.70
Rhoifolin	2.58 ± 0.025	0.05 ± 0.005	------	------	------
Neodiosmin	3.33 ± 0.02	0.08 ± 0.008	------	------	------
Isorhoifolin	------	0.07 ± 0.007	------	------	------
Sinensetin	------	0.37 ± 0.04	------	1.85 ± 0.11	1.03 ± 0.07
Tangeretin	23.37 ± 0.42	0.04 ± 0.004	1.04 ± 0.07	1.32 ± 0.12	0.61 ± 0.08
Chrysoeriol 7-*O*- neohesperidoside -4′-*O*- glucoside	------	------	------	0.70 ± 0.03	------
Nobiletin	------	0.33 ± 0.19	1.04 ± 0.10	1.25 ± 0.17	0.84 ± 0.05
Apigenin	9.67 ± 0.12	------	------	------	------
Heptamethoxy-flavone	------	0.08 ± 0.06	------	------	0.07 ± 0.007
Quercetogetin	------	------	------	------	0.06 ± 0.006
	[31]	[22]	[30]	[30]	[30]

## References

[1] United States Department of Agriculture. https://www.fas.usda.gov/psdonline/circulars/citrus.pdf.

[2] Moore G.A. (2001). Oranges and lemons: Clues to the taxonomy of Citrus from molecular markers. Trends Genet..

[3] Velasco R., Licciardello C. (2014). A genealogy of the citrus family. Nat. Biotechnol..

[4] Mabberley D.J. (1997). A classification for edible *Citrus* (Rutaceae). Telopea.

[5] Calabrese F. (1973). Considerazioni sulla classificazione botanica delle Aurantioideae (Rutaceae). Webbia J. Plant Taxon. Geogr..

[6] Wu G.A., Terol J., Ibanez V., López-García A., Pérez-Román E., Borredá C., Domingo C., Tadeo F.R., Carbonell-Caballero J., Alonso R. (2018). Genomics of the origin and evolution of *Citrus*. Nature.

[7] Panche A.N., Diwan A.D., Chandra S.R. (2016). Flavonoids: An overview. J. Nutr. Sci..

[8] Del Rio D., Rodriguez-Mateos A., Spencer J.P.E., Tognolini M., Borges G., Crozier A. (2013). Dietar (Poly) phenolics in Human Health: Structures, Bioavailability, and Evidence of Protective Effects Against Chronic Diseases. Antioxid. Redox Signal..

[9] Strack D., Wray V., Harborne J.B. (1992). Anthocyanins. The Flavonoids: Advances in Research since 1986.

[10] Stafford H. (1990). Flavonoid Metabolism.

[11] Dudek B., Warskulat A.-C., Schneider B. (2016). The occurrence of flavonoids and related compounds in flower sections of Papaver nudicaule. Plants.

[12] Tung Y.-C., Chou Y.-C., Hung W.-L., Cheng A.-C., Yu R.-C., Ho C.-T., Pan M.-H. (2019). Polymethoxyflavones: Chemistry and Molecular Mechanisms for Cancer Prevention and Treatment. Curr. Pharmacol. Rep..

[13] Barreca D., Gattuso G., Bellocco E., Calderaro A., Trombetta D., Smeriglio A., Laganà G., Daglia M., Meneghini S., Nabavi S.M. (2017). Flavanones: Citrus phytochemical with health-promoting properties. BioFactors.

[14] Barreca D., Bellocco E., Leuzzi U., Gattuso G. (2014). First evidence of *C*- and *O*-glycosyl flavone in blood orange (*Citrus sinensis* (L.) Osbeck) juice and their influence on antioxidant properties. Food Chem..

[15] Barreca D., Gattuso G., Laganà G., Leuzzi U., Bellocco E. (2016). *C*- and *O*-glycosyl flavonoids in Sanguinello and Tarocco blood orange (*Citrus sinensis* (L.) Osbeck) juice: Identification and influence on antioxidant properties and acetylcholinesterase activity. Food Chem..

[16] Barreca D., Bellocco E., Caristi C., Leuzzi U., Gattuso G. (2011). Kumquat (*Fortunella japonica* Swingle) juice: Flavonoid distribution and antioxidant properties. Food Res. Int..

[17] Gattuso G., Barreca D. (2012). Juice analysis in *Citrus*: Latest developments. Advances in Citrus Nutrition.

[18] Wang S., Yang C., Tu H., Zhou J., Liu X., Cheng Y., Luo J., Deng X., Zhang H., Xu J. (2017). Characterization and Metabolic Diversity of Flavonoids in *Citrus* Species. Sci. Rep..

[19] Nogata Y., Sakamoto K., Shiratsuchi H., Ishii T., Yano M., Ohta H. (2006). Flavonoid composition of fruit tissues of citrus species. Biosci. Biotechnol. Biochem..

[20] Barreca D., Bellocco E., Leuzzi U., Gattuso G. (2014). Flavonoid *C*-glycosides in *Citrus* Juices from Southern Italy: Distribution and Influence on the Antioxidant Activity. Instrumental Methods for the Analysis and Identification of Bioactive Molecules.

[21] Barreca D., Bellocco E., Ficarra S., Laganà G., Galtieri A., Tellone E., Gattuso G. (2018). Analysis of C-Glycosyl Flavones and 3-Hydroxy-3-methylglutaryl-glycosyl Derivatives in Blood Oranges (*Citrus sinensis* (L.) Osbeck) Juices and Their Influence on Biological Activity. Advances in Plant Phenolics: From Chemistry to Human Health.

[22] Gattuso G., Barreca D., Gargiulli C., Leuzzi U., Caristi C. (2007). Flavonoid Composition of Citrus Juices. Molecules.

[23] Ledesma-Escobar C.A., Priego-Capote F., Luque de Castro M.D. (2016). Comparative Study of the Effect of Sample Pretreatment and Extraction on the Determination of Flavonoids from Lemon (*Citrus limon*). PLoS ONE.

[24] Gattuso G., Barreca D., Caristi C., Gargiulli C., Leuzzi U. (2007). Distribution of Flavonoids and Furocoumarins in Juices from Cultivars of *Citrus bergamia* Risso. J. Agric. Food Chem..

[25] Barreca D., Bellocco E., Caristi C., Leuzzi U., Gattuso G. (2010). Flavonoid Composition and Antioxidant Activity of Juices from Chinotto (*Citrus* × *myrtifolia* Raf.) Fruits at Different Ripening Stages. J. Agric. Food Chem..

[26] Barreca D., Bellocco E., Caristi C., Leuzzi U., Gattuso G. (2011). Distribution of C- and O-glycosyl flavonoids, (3-hydroxy-3-methylglutaryl)glycosyl flavanones and furocoumarins in *Citrus aurantium* L. juice. Food Chem..

[27] Barreca D., Bellocco E., Caristi C., Leuzzi U., Gattuso G. (2011). Flavonoid profile and radical-scavenging activity of Mediterranean sweet lemon (*Citrus limetta* Risso) juice. Food Chem..

[28] Barreca D., Bellocco E., Caristi C., Leuzzi U., Gattuso G. (2011). Elucidation of the flavonoid and furocoumarin composition and radical-scavenging activity of green and ripe chinotto (*Citrus myrtifolia* Raf.) fruit tissues, leaves and seeds. Food Chem..

[29] Barreca D., Bisignano C., Ginestra G., Bisignano G., Bellocco E., Leuzzi U., Gattuso G. (2013). Polymethoxylated, *C*- and *O*-glycosyl flavonoids in Tangelo (*C. reticulata* × *C. paradisi*) juice and their influence on antioxidant properties. Food Chem..

[30] Theile D., Hohmann N., Kiemel D., Gattuso G., Barreca D., Mikus G., Haefeli W.E., Schwenger V., Weiss J. (2017). Clementine juice has the potential for drug interactions–In vitro comparison with grapefruit and mandarin juice. Eur. J. Pharm. Sci..

[31] Smeriglio A., Cornara L., Denaro M., Barreca D., Burlando B., Xiao J., Trombetta D. (2019). Antioxidant and cytoprotective activities of an ancient Mediterranean citrus (*Citrus lumia* Risso) albedo extract: Microscopic observations and polyphenol characterization. Food Chem..

[32] Hohmann N., Mikus G., Haefeli W.E., Schwenger V., Gattuso G., Barreca D., Weiss J. (2019). A follow-up report on potential drug interactions with clementines: Two single case experiments show no effect on CYP3A-dependent midazolam clearance. Eur. J. Pharm. Sci..

[33] Barreca D., Bellocco E., Caristi C., Leuzzi U., Gattuso G. (2013). Flavonoid and Antioxidant Properties of Fruits Belonging to the Annona and Citrus Genera. Tropical and Subtropical Fruit: Flavors, Color, and Health Benefits.

[34] Barreca D., Bellocco E., Caristi C., Leuzzi U., Gattuso G. (2012). Flavonoids and Furocoumarins in Bergamot, Myrtle-leaved Orange and Sour Orange Juices: Distribution and Properties. Emerging Trends in Dietary Components for Preventing and Combating Disease.

[35] Barreca D., Bellocco E., Caristi C., Leuzzi U., Gattuso G. (2012). Flavonoid distribution in neglected Citrus species grown in the Mediterranean basin. Flavonoids: Dietary Sources, Properties and Health Benefits.

[36] Manach C., Williamson G., Morand C., Scalbert A., Remesy C. (2005). Bioavailability and bioefficacy of polyphenols in humans. I. Review of 97 bioavailability studies. Am. J. Clin. Nutr..

[37] Hostetler G.L., Ralston R.A., Schwartz S.J. (2017). Flavones: Food Sources, Bioavailability, Metabolism, and Bioactivity. Adv. Nutr..

[38] Wang M., Firrman J., Liu L., Yam K. (2019). A Review on Flavonoid Apigenin: Dietary Intake, ADME, Antimicrobial Effects, and Interactions with Human Gut Microbiota. BioMed Res. Int..

[39] Singh G., Verma A.K., Kumar V. (2016). Catalytic properties, functional attributes and industrial applications of β-glucosidases. Biotech.

[40] Cione E., La Torre C., Cannataro R., Caroleo M.C., Plastina P., Gallelli L. (2019). Quercetin, Epigallocatechin Gallate, Curcumin, and Resveratrol: From Dietary Sources to Human MicroRNA Modulation. Molecules.

[41] Shi J., Zheng H., Yu J., Zhu L., Yan T., Wu P., Lu L., Wang Y., Hu M., Liu Z. (2016). SGLT-1 Transport and Deglycosylation inside Intestinal Cells Are Key Steps in the Absorption and Disposition of Calycosin-7-*O*-β-d-Glucoside in Rats. Drug Metab. Dispos..

[42] Gonzales G.B. (2017). In vitro bioavailability and cellular bioactivity studies of flavonoids and flavonoid-rich plant extracts: Questions, considerations and future perspectives. Proc. Nutr. Soc..

[43] Lam P.Y., Liu H., Lo C. (2015). Completion of Tricin Biosynthesis Pathway in Rice: Cytochrome P450 75B4 Is a Unique Chrysoeriol 5’-Hydroxylase. Plant Physiol..

[44] Hung W.L., Chang W.S., Lu W.C., Wei G.J., Wang Y., Ho C.T., Hwang L.S. (2018). Pharmacokinetics, bioavailability, tissue distribution and excretion of tangeretin in rat. J. Food Drug Anal..

[45] Burapan S., Kim M., Han J. (2017). Demethylation of polymethoxyflavones by human gut bacterium, Blautia sp. MRG-PMF1. J. Agric. Food Chem..

[46] Kim M., Kim N., Han J. (2014). Metabolism of Kaempferia parviflora polymethoxyflavones by human intestinal bacterium Bautia sp. MRG-PMF1. J. Agric. Food Chem..

[47] Patel K., Gadewar M., Tahilyani V., Patel D.K. (2013). A review on pharmacological and analytical aspects of diosmetin: A concise report. Chin. J. Integr. Med..

[48] Silvestro L., Tarcomnicu I., Dulea C., Attili N.R., Ciuca V., Peru D., Rizea Savu S. (2013). Confirmation of diosmetin 3-*O*-glucuronide as major metabolite of diosmin in humans, using micro-liquid-chromatography-mass spectrometry and ion mobility mass spectrometry. Anal. Bioanal. Chem..

[49] Chen X., Xu L., Guo S., Wang Z., Jiang L., Wang F., Zhang J., Liu B. (2019). Profiling and comparison of the metabolites of diosmetin and diosmin in rat urine, plasma and feces using UHPLC-LTQ-Orbitrap MS(n). J. Chromatogr. B Anal. Technol. Biomed Life Sci..

[50] Szeleszczuk L., Pisklak D.M., Zielinska-Pisklak M., Wawer I. (2017). Spectroscopic and structural studies of the diosmin monohydrate and anhydrous diosmin. Int. J. Pharm..

[51] Russo R., Chandradhara D., De Tommasi N. (2018). Comparative Bioavailability of Two Diosmin Formulations after Oral Administration to Healthy Volunteers. Molecules.

[52] Bush R., Comerota A., Meissner M., Raffetto J.D., Hahn S.R., Freeman K. (2017). Recommendations for the medical management of chronic venous disease: The role of Micronized Purified Flavanoid Fraction (MPFF). Phlebology.

[53] Breiter T., Laue C., Kressel G., Gröll S., Engelhardt U.H., Hahn A. (2011). Bioavailability and antioxidant potential of rooibos flavonoids in humans following the consumption of different rooibos formulations. Food Chem..

[54] Tripoli E., La Guardia M., Giammanco S., Di Majo D., Giammanco M. (2007). Citrus flavonoids: Molecular structure, biological activity and nutritional properties: A review. Food Chem..

[55] Kumar S., Pandey A.K. (2013). Chemistry and Biological Activities of Flavonoids: An Overview. Sci. World J..

[56] Salehi B., Venditti A., Sharifi-Rad M., Kręgiel D., Sharifi-Rad J., Durazzo A., Lucarini M., Santini A., Souto E.B., Novellino E. (2019). The Therapeutic Potential of Apigenin. Int. J. Mol. Sci..

[57] López-Lázaro M. (2009). Distribution and biological activities of the flavonoid luteolin. Mini Rev. Med. Chem..

[58] Kritas S.K., Saggini A., Varvara G., Murmura G., Caraffa A., Antinolfi P., Toniato E., Pantalone A., Neri G., Frydas S. (2013). Luteolin inhibits mast cell-mediated allergic inflammation. J. Biol. Regul. Homeost. Agents.

[59] Castro-Vazquez L., Alañón M.E., Rodríguez-Robledo V., Pérez-Coello M.S., Hermosín-Gutierrez I., Díaz-Maroto M.C., Jordán J., Galindo M.F., Arroyo-Jiménez M. (2016). Bioactive Flavonoids, Antioxidant Behaviour, and Cytoprotective Effects of Dried Grapefruit Peels (*Citrus paradisi* Macf.). Oxidative Med. Cell Longev..

[60] Silva M.M., Santos M.R., Caroço G., Rocha R., Justino G., Mira L. (2002). Structure-antioxidant activity relationships of flavonoids: A re-examination. Free Radic. Res..

[61] van Acker S.A., van den Berg D.J., Tromp M.N., Griffioen D.H., van Bennekom W.P., van der Vijgh W.J., Bast A. (1996). Structural aspects of antioxidant activity of flavonoids. Free Radic. Biol. Med..

[62] Pietta P.G. (2000). Flavonoids as antioxidants. J. Nat. Prod..

[63] Galleano M., Verstraeten S.V., Oteiza P.I., Fraga C.G. (2010). Antioxidant actions of flavonoids: Thermodynamic and kinetic analysis. Arch. Biochem. Biophys..

[64] Bellocco E., Barreca D., Laganà G., Leuzzi U., Tellone E., Kotyk A., Galtieri A. (2009). Influence of L-rhamnosyl-D-glucosyl derivatives on properties and biological interaction of flavonoids. Mol. Cell. Biochem..

[65] Barreca D., Laganà G., Tellone E., Ficarra S., Leuzzi U., Galtieri A., Bellocco E. (2009). Influences of flavonoids on erythrocyte membrane and metabolic implication through anionic exchange modulation. J. Membr. Biol..

[66] Barreca D., Laganà G., Bruno G., Magazù S., Bellocco E. (2013). Diosmin binding to human serum albumin and its preventive action against degradation due to oxidative injuries. Biochimie.

[67] Rice-Evans C.A., Miller N.J., Paganga G. (1996). Structure-antioxidant activity relationships of flavonoids and phenolic acids. Free Radic. Biol. Med..

[68] Zielińska D., Zielińskib H. (2011). Antioxidant activity of flavone *C*-glucosides determined by updated analytical strategies. Food Chem..

[69] Wolfe K.L., Liu R.H. (2008). Structure-activity relationships of flavonoids in the cellular antioxidant activity assay. J. Agric. Food Chem..

[70] Moalin M., van Strijdonck G.P.F., Beckers M., Hagemen G.J., Borm P.J., Bast A., Haenen G.R. (2011). A planar conformation and the hydroxyl groups in the B and C rings play a pivotal role in the antioxidant capacity of quercetin and quercetin derivatives. Molecules.

[71] Furusawa M., Tanaka T., Ito T., Nishikawa A., Yamazaki N., Nakaya K.-I., Matsuura N., Tsuchiya H., Nagayama M., Iinuma M. (2005). Antioxidant activity of hydroflavonoids. J. Health Sci..

[72] Bors W., Heller W., Michel C., Saran M. (1990). Flavonoids as antioxidants: Determination of radical scavenging efficiencies. Methods Enzymol..

[73] Arora A., Nair M.G., Strasburg G.M. (1998). Structure-activity relationships for antioxidant activities of a series of flavonoids in a liposomal system. Free Radic. Biol Med..

[74] Cao G., Sofic E., Prior R.L. (1997). Antioxidant and prooxidant behavior of flavonoids: Structure-activity relationships. Free Radic. Biol. Med..

[75] de Souza V.T., de Franco É.P., de Araújo M.E., Messias M.C., Priviero F.B., Frankland Sawaya A.C., de Oliveira Carvalho P. (2016). Characterization of the antioxidant activity of aglycone and glycosylated derivatives of hesperetin: An in vitro and in vivo study. J. Mol. Recognit..

[76] Kimata M., Shichijo M., Miura T., Serizawa I., Inagaki N., Nagai H. (2000). Effects of luteolin, quercetin and baicalein on immunoglobulin E-mediated mediator release from human cultured mast cells. Clin. Exp. Allergy.

[77] Weng Z., Patel A.B., Panagiotidou S., Theoharides T.C. (2015). The novel flavone tetramethoxyluteolin is a potent inhibitor of human mast cells. J. Allergy Clin. Immunol..

[78] Zhu S., Xu T., Luo Y., Zhang Y., Xuan H., Ma Y., Zhu H. (2017). Luteolin enhances sarcoplasmic reticulum Ca^2+^-ATPase activity through p38 MAPK signaling thus improving rat cardiac function after ischemia/reperfusion. Cell. Physiol. Biochem..

[79] Funakoshi-Tago M., Nakamura K., Tago K., Mashino T., Kasahara T. (2011). Anti-inflammatory activity of structurally related flavonoids, Apigenin, Luteolin and Fisetin. Int. Immunopharmacol..

[80] Mandalari G., Bisignano C., D’Arrigo M., Ginestra G., Arena A., Tomaino A., Wickham M.S. (2010). Antimicrobial potential of polyphenols extracted from almond skins. Lett. Appl. Microbiol..

[81] Bisignano C., Filocamo A., La Camera E., Zummo S., Fera M.T., Mandalari G. (2013). Antibacterial activities of almond skins on cagA-positive and-negative clinical isolates of Helicobacter pylori. BMC Microbiol..

[82] Arena A., Bisignano C., Stassi G., Filocamo A., Mandalari G. (2015). Almond Skin Inhibits HSV-2 Replication in Peripheral Blood Mononuclear Cells by Modulating the Cytokine Network. Molecules.

[83] Bisignano C., Mandalari G., Smeriglio A., Trombetta D., Pizzo M.M., Pennisi R., Sciortino M.T. (2017). Almond Skin Extracts Abrogate HSV-1 Replication by Blocking Virus Binding to the Cell. Virus.

[84] Mandalari G., Bennett R.N., Bisignano G., Trombetta D., Saija A., Faulds C.B., Gasson M.J., Narbad A. (2007). Antimicrobial activity of flavonoids extracted from bergamot (*Citrus bergamia* Risso) peel, a byproduct of the essential oil industry. J. Appl. Microbiol..

[85] Filocamo A., Bisignano C., Ferlazzo N., Cirmi S., Mandalari G., Navarra M. (2015). In vitro effect of bergamot (*Citrus bergamia*) juice against cagA-positive and-negative clinical isolates of Helicobacter pylori. BMC Complementary Altern. Med..

[86] Uckoo R.M., Jayaprakasha G.K., Vikram A., Patil B.S. (2015). Polymethoxyflavones Isolated from the Peel of Miaray Mandarin (*Citrus miaray*) Have Biofilm Inhibitory Activity in Vibrio harveyi. J. Agric. Food Chem..

[87] Johann S., Oliveira V.L., Pizzolatti M.G., Schripsema J., Braz-Filho R., Branco A., Smânia A. (2007). Antimicrobial activity of wax and hexane extracts from Citrus spp. peels. Memórias Inst. Oswaldo Cruz.

[88] Murakami A., Nakamura Y., Ohto Y., Yano M., Koshiba T., Koshimizu K., Tokuda H., Nishino H., Ohigashi H. (2000). Suppressive effects of citrus fruits on free radical generation and nobiletin, an anti-inflammatory polymethoxyflavonoid. Biofactors.

[89] Chan B.C., Ip M., Gong H., Lui S.L., See R.H., Jolivalt C., Fung K.P., Leung P.C., Reiner N.E., Lau C.B. (2013). Synergistic effects of diosmetin with erythromycin against ABC transporter over-expressed methicillin-resistant Staphylococcus aureus (MRSA) RN4220/pUL5054 and inhibition of MRSA pyruvate kinase. Phytomedicine.

[90] Ernawita Wahyuono R.A., Hesse J., Hipler U.C., Elsner P., Böhm V. (2017). In Vitro Lipophilic Antioxidant Capacity, Antidiabetic and Antibacterial Activity of Citrus Fruits Extracts from Aceh, Indonesia. Antioxidants.

[91] Adamczak A., Ożarowski M., Karpiński T.M. (2019). Antibacterial Activity of Some Flavonoids and Organic Acids Widely Distributed in Plants. J. Clin. Med..

[92] Echeverría J., Opazo J., Mendoza L., Urzúa A., Wilkens M. (2017). Structure-Activity and Lipophilicity Relationships of Selected Antibacterial Natural Flavones and Flavanones of Chilean Flora. Molecules.

[93] Xu J.J., Wu X., Li M.M., Li G.Q., Yang Y.T., Luo H.J., Huang W.H., Chung H.Y., Ye W.C., Wang G.C. (2014). Antiviral activity of polymethoxylated flavones from “Guangchenpi”, the edible and medicinal pericarps of citrus reticulata ‘Chachi’. J. Agric. Food Chem..

[94] Xu J.J., Liu Z., Tang W., Wang G.C., Chung H.Y., Liu Q.Y., Zhuang L., Li M.M., Li Y.L. (2015). Tangeretin from Citrus reticulate Inhibits Respiratory Syncytial Virus Replication and Associated Inflammation in Vivo. J. Agric. Food Chem..

[95] Tang K., He S., Zhang X., Guo J., Chen Q., Yan F., Banadyga L., Zhu W., Qiu X., Guo Y. (2018). Tangeretin, an extract from Citrus peels, blocks cellular entry of arenaviruses that cause viral hemorrhagic fever. Antivir. Res..

[96] Martin-Benlloch X., Haid S., Novodomska A., Rominger F., Pietschmann T., Davioud-Charvet E., Elhabiri M. (2019). Physicochemical Properties Govern the Activity of Potent Antiviral Flavones. ACS Omega.

[97] Sadati S.M., Gheibi N., Ranjbar S., Hashemzadeh M.S. (2019). Docking study of flavonoid derivatives as potent inhibitors of influenza H1N1 virus neuramidases. Biomed. Rep..

[98] Chahar M.K., Sharma N., Dobhal M.P., Joshi Y.C. (2011). Flavonoids: A versatile source of anticancer drugs. Pharmacogn. Rev..

[99] Kanadaswami C., Lee L.-T., Lee P.-P.H., Hwang J.-J., Ke F.-C., Huang Y.-T., Lee M.-T. (2005). The Antitumor Activities of Flavonoids. In Vivo.

[100] Takagaki N., Sowa Y., Oki T., Nakanishi R., Yogosawa S., Sakai T. (2005). Apigenin induces cell cycle arrest and p21/WAF1 expression in a p53-independent pathway. Int. J. Oncol..

[101] Maggioni D., Garavello W., Rigolio R., Pignataro L., Gaini R., Nicolini G. (2013). Apigenin impairs oral squamous cell carcinoma growth in vitro inducing cell cycle arrest and apoptosis. Int. J. Oncol..

[102] Iizumi Y., Oishi M., Taniguchi T., Goi W., Sowa Y., Sakai T. (2013). The flavonoid apigenin downregulates CDK1 by directly targeting ribosomal protein S9. PLoS ONE.

[103] Seo H.S., Ku J.M., Choi H.S., Woo J.K., Jang B.H., Shin Y.C., Ko S.G. (2014). Induction of caspase-dependent apoptosis by apigenin by inhibiting STAT3 signaling in HER2-overexpressing MDA-MB-453 breast cancer cells. Anticancer Res..

[104] Seo H.S., Choi H.S., Kim S.R., Choi Y.K., Woo S.M., Shin I., Woo J.K., Park S.Y., Shin Y.C., Ko S.K. (2012). Apigenin induces apoptosis via extrinsic pathway, inducing p53 and inhibiting STAT3 and NF-kB signalling in HER2-overexpressing breast cancer cells. Mol. Cell. Biochem..

[105] Karmakar S., Davis K.A., Choudhury S.R., Deeconda A., Banik N.L., Ray S.K. (2009). Bcl-2 inhibitor and apigenin worked synergistically in human malignant neuroblastoma cell lines and increased apoptosis with activation of extrinsic and intrinsic pathways. Biochem. Biophys. Res. Commun..

[106] Peng Q., Deng Z., Pan H., Gu L., Liu O., Tang Z. (2017). Mitogen-activated protein kinase signaling pathway in oral cancer. Oncol. Lett..

[107] Xie Y., Liang D., Wu Q., Chen X., Buabeid M.A., Wang Y. (2019). A System-Level Investigation into the Mechanisms of Apigenin against Inflammation. Nat. Prod. Commun..

[108] Bauer D., Mazzio E., Soliman K.F.A. (2019). Whole Transcriptomic Analysis of Apigenin on TNFα Immuno-activated MDA-MB-231 Breast Cancer Cells. Cancer Genom. Proteom..

[109] Lee H.H., Jung J., Moon A., Kang H., Cho H. (2019). Antitumor and Anti-Invasive Effect of Apigenin on Human Breast Carcinoma through Suppression of IL-6 Expression. Int. J. Mol. Sci..

[110] Sinha S., Patel S., Athar M., Vora J., Chhabria M.T., Jha P.C., Shrivastava N. (2019). Structure-based identification of novel sirtuin inhibitors against triple negative breast cancer: An in silico and in vitro study. Int. J. Biol. Macromol..

[111] Pace E., Di Vincenzo S., Di Salvo E., Genovese S., Dino P., Sangiorgi C., Ferraro M., Gangemi S. (2019). MiR-21 upregulation increases IL-8 expression and tumorigenesis program in airway epithelial cells exposed to cigarette smoke. J. Cell. Physiol..

[112] Fan X., Bai J., Zhao S., Hu M., Sun Y., Wang B., Ji M., Jin J., Wang X., Hu J. (2019). Evaluation of inhibitory effects of flavonoids on breast cancer resistance protein (BCRP): From library screening to biological evaluation to structure activity relationship. Toxicol. In Vitro.

[113] Chen X., Xu H., Yu X., Wang X., Zhu X., Xu X. (2019). Apigenin inhibits in vitro and in vivo tumorigenesis in cisplatin-resistant colon cancer cells by inducing autophagy, programmed cell death and targeting m-TOR/PI3K/Akt signalling pathway. JBUON.

[114] Zhan Y., Wang Y., Qi M., Liang P., Ma Y., Li T., Li H., Dai C., An Z., Qi Y. (2019). BH3 mimetic ABT-263 enhances the anticancer effects of apigenin in tumor cells with activating EGFR mutation. Cell. Biosci..

[115] Qiu J.G., Wang L., Liu W.J., Wang J.F., Zhao E.J., Zhou F.M., Ji X.B., Wang L.H., Xia Z.K., Wang W. (2019). Apigenin Inhibits IL-6 Transcription and Suppresses Esophageal Carcinogenesis. Front. Pharmacol..

[116] Ahmed S., Khan H., Fratantonio D., Hasan M.M., Sharifi S., Fathi N., Ullah H., Rastrelli L. (2019). Apoptosis induced by luteolin in breast cancer: Mechanistic and therapeutic Perspectives. Phytomedicine.

[117] Imran M., Rauf A., Abu-Izneid T., Nadeem M., Shariati M.A., Khan I.A., Imran A., Orhan I.E., Rizwan M., Atif M. (2019). Luteolin, a flavonoid, as an anticancer agent: A review. Biomed. Pharmacother..

[118] Avila-Carrasco L., Majano P., Sánchez-Toméro J.A., Selgas R., López-Cabrera M., Aguilera A., González Mateo G. (2019). Natural Plants Compounds as Modulators of Epithelial-to-Mesenchymal Transition. Front. Pharmacol..

[119] Lee J., Park S.H., Lee J., Chun H., Choi M.K., Yoon J.H., Pham T.H., Kim K.H., Kwon T., Ryu H.W. (2019). Differential effects of luteolin and its glycosides on invasion and apoptosis in MDA-MB-231 triple-negative breast cancer cells. EXCLI J..

[120] Yao X., Jiang W., Yu D., Yan Z. (2019). Luteolin inhibits proliferation and induces apoptosis of human melanoma cells in vivo and in vitro by suppressing MMP-2 and MMP-9 through the PI3K/AKT pathway. Food Funct..

[121] Huang L., Jin K., Lan H. (2019). Luteolin inhibits cell cycle progression and induces apoptosis of breast cancer cells through downregulation of human telomerase reverse transcriptase. Oncol. Lett..

[122] Aryappalli P., Shabbiri K., Masad R.J., Al-Marri R.H., Haneefa S.M., Mohamed Y.A., Arafat K., Attoub S., Cabral-Marques O., Ramadi K.B. (2019). Inhibition of Tyrosine-Phosphorylated STAT3 in Human Breast and Lung Cancer Cells by Manuka Honey is Mediated by Selective Antagonism of the IL-6 Receptor. Int. J. Mol. Sci..

[123] Yu Q., Zhang M., Ying Q., Xie X., Yue S., Tong B., Wei Q., Bai Z., Ma L. (2019). Decrease of AIM2 mediated by luteolin contributes to non-small cell lung cancer treatment. Cell Death Dis..

[124] Fan J.J., Wen-Hsien Hsu W.H., Lee K.H., Chen K.C., Lin C.W., Lee Y.L.A., Ko T.P., Lee L.A., Lee M.T., Chang M.S. (2019). Dietary Flavonoids Luteolin and Quercetin Inhibit Migration and Invasion of Squamous Carcinoma through Reduction of Src/Stat3/S100A7. Signal. Antioxid..

[125] Gao G., Ge R., Li Y., Liu S. (2019). Luteolin exhibits anti-breast cancer property through up-regulating miR-203. Artif. Cells Nanomed. Biotechnol..

[126] Yao Y., Rao C., Zheng G., Wang S. (2019). Luteolin suppresses colorectal cancer cell metastasis via regulation of the miR-384/pleiotrophin axis. Oncol. Rep..

[127] Ferino A., Rapozzi V., Xodo L.E. (2020). The ROS-KRAS-Nrf2 axis in the control of the redox homeostasis and the intersection with survival-apoptosis pathways: Implications for photodynamic therapy. J. Photochem. Photobiol. B.

[128] Kang K.A., Piao M.J., Hyun Y.J., Zhen A.X., Cho S.J., Ahn M.J., Yi J.M., Hyun J.W. (2019). Luteolin promotes apoptotic cell death via upregulation of Nrf2 expression by DNA demethylase and the interaction of Nrf2 with p53 in human colon cancer cells. Exp. Mol. Med..

[129] Soliman N.A., Abd-Ellatif R.N., ELSaadany A.A., Shalaby S.M., Bedeer A.E. (2019). Luteolin and 5-flurouracil act synergistically to induce cellular weapons in experimentally induced Solid Ehrlich Carcinoma: Realistic role of P53; a guardian fights in a cellular battle. Chem. Biol. Interact..

[130] Wu C., Xu Q., Chen X., Liu J. (2019). Delivery luteolin with folacin-modified nanoparticle for glioma therapy. Int. J. Nanomed..

[131] Androutsopoulos V.P., Mahale S., Arroo R.R., Potter G. (2009). Anticancer effects of the flavonoid diosmetin on cell cycle progression and proliferation of MDA-MB 468 breast cancer cells due to CYP1 activation. Oncol. Rep..

[132] Androutsopoulos V., Wilsher N., Arroo R.R., Potter G.A. (2009). Bioactivation of the phytoestrogen diosmetin by CYP1 cytochromes P450. Cancer Lett..

[133] Androutsopoulos V.P., Spandidos D.A. (2013). The flavonoids diosmetin and luteolin exert synergistic cytostatic effects in human hepatoma HepG2 cells via CYP1A-catalyzed metabolism, activation of JNK and ERK and P53/P21 up-regulation. J. Nutr. Biochem..

[134] Wang C., Li S., Ren H., Sheng Y., Wang T., Li M., Zhou Q., He H., Liu C. (2019). Anti-Proliferation and Pro-Apoptotic Effects of Diosmetin via Modulating Cell Cycle Arrest and Mitochondria-Mediated Intrinsic Apoptotic Pathway in MDA-MB-231 Cells. Med. Sci. Monit..

[135] Koosha S., Mohamed Z., Sinniah A., Alshawsh M.A. (2019). Investigation into the Molecular mechanisms underlying the Anti-proliferative and Anti-tumorigenesis activities of Diosmetin against HCT-116 Human Colorectal Cancer. Sci. Rep..

[136] Oak C., Khalifa A.O., Isali I., Bhaskaran N., Walker E., Shukla S. (2018). Diosmetin suppresses human prostate cancer cell proliferation through the induction of apoptosis and cell cycle arrest. Int. J. Oncol..

[137] Xu Z., Yan Y., Xiao L., Dai S., Zeng S., Qian L., Wang L., Yang X., Xiao Y., Gong Z. (2017). Radiosensitizing effect of diosmetin on radioresistant lung cancer cells via Akt signaling pathway. PLoS ONE.

[138] Liu B., Shi Y., Peng W., Zhang Q., Liu J., Chen N., Zhu R. (2016). Diosmetin induces apoptosis by upregulating p53 via the TGF-β signal pathway in HepG2 hepatoma cells. Mol. Med. Rep..

[139] Qiao J., Liu J., Jia K., Li N., Liu B., Zhang Q., Zhu R. (2016). Diosmetin triggers cell apoptosis by activation of the p53/Bcl-2 pathway and inactivation of the Notch3/NF-κB pathway in HepG2 cells. Oncol. Lett..

[140] Liu J., Ren H., Liu B., Zhang Q., Li M., Zhu R. (2016). Diosmetin inhibits cell proliferation and induces apoptosis by regulating autophagy via the mammalian target of rapamycin pathway in hepatocellular carcinoma HepG2 cells. Oncol. Lett..

[141] Roma A., Rota S.G., Spagnuolo P.A. (2018). Diosmetin Induces Apoptosis of Acute Myeloid Leukemia. Cells Mol. Pharm..

[142] Chen X., Wu Q., Chen Y., Zhang J., Li H., Yang Z., Yang Y., Deng Y., Zhang L., Liu B. (2019). Diosmetin induces apoptosis and enhances the chemotherapeutic efficacy of paclitaxel in non-small cell lung cancer cells via Nrf2 inhibition. Br. J. Pharmacol..

[143] Liu J., Wen X., Liu B., Zhang Q., Zhang J., Miao H., Zhu R. (2016). Diosmetin inhibits the metastasis of hepatocellular carcinoma cells by downregulating the expression levels of MMP-2 and MMP-9. Mol. Med. Rep..

[144] Koosha S., Mohamed Z., Sinniah A., Alshawsh M.A. (2019). Evaluation of Anti-Tumorigenic Effects of Diosmetin against Human Colon Cancer Xenografts in Athymic Nude Mice. Molecules.

[145] Choi J., Lee D.H., Park S.Y., Seol J.W. (2019). Diosmetin inhibits tumor development and block tumor angiogenesis in skin cancer. Biomed. Pharmacother..

[146] Takemura H., Uchiyama H., Ohura T., Sakakibara H., Kuruto R., Amagai T., Shimoi K. (2010). A methoxyflavonoid, chrysoeriol, selectively inhibits the formation of a carcinogenic estrogen metabolite in MCF-7 breast cancer cells. J. Steroid Biochem. Mol. Biol..

[147] Baier A., Galicka A., Nazaruk J., Szyszka R. (2017). Selected flavonoid compounds as promising inhibitors of protein kinase CK2α and CK2α’, the catalytic subunits of CK2. Phytochemistry.

[148] Tan K.W., Li Y., Paxton J.W., Birch N.P., Scheepens A. (2013). Identification of novel dietary phytochemicals inhibiting the efflux transporter breast cancer resistance protein (BCRP/ABCG2). Food Chem..

[149] Lai S., Chen J.N., Huang H.W., Zhang X.Y., Jiang H.L., Li W., Wang P.L., Wang J., Liu F.N. (2018). Structure activity relationships of chrysoeriol and analogs as dual c-Met and VEGFR2 tyrosine kinase inhibitors. Oncol. Rep..

[150] Zhang Y., Li Z., Min Q., Palida A., Zhang Y., Tang R., Chen L., Li H. (2018). 8-Chrysoeriol, as a potential BCL-2 inhibitor triggers apoptosis of SW1990 pancreatic cancer cells. Bioorg. Chem..

[151] Amrutha K., Nanjan P., Shaji S.K., Sunilkumar D., Subhalakshmi K., Rajakrishna L., Banerji A. (2014). Discovery of lesser known flavones as inhibitors of NF-κB signaling in MDA-MB-231 breast cancer cells--A SAR study. Bioorg. Med. Chem. Lett..

[152] Yang Y., Zhou X., Xiao M., Hong Z., Gong Q., Jiang L., Zhou J. (2010). Discovery of chrysoeriol, a PI3K-AKT-mTOR pathway inhibitor with potent antitumor activity against human multiple myeloma cells in vitro. J. Huazhong Univ. Sci. Technol. Med. Sci..

[153] Wei W., He J., Ruan H., Wang Y. (2019). In vitro and in vivo cytotoxic effects of chrysoeriol in human lung carcinoma are facilitated through activation of autophagy, sub-G1/G0 cell cycle arrest, cell migration and invasion inhibition and modulation of MAPK/ERK signaling pathway. J. BUON.

